# Case Report: Concurrent atrial and ventricular septal defect in a young Sphynx cat

**DOI:** 10.3389/fvets.2025.1684236

**Published:** 2025-11-19

**Authors:** Jihoon Lee, Byungkwan Oh, Sang-Ik Oh, Bumseok Kim, Hyungjin Park, Kichang Lee, Hakyoung Yoon

**Affiliations:** 1 Department of Veterinary Medical Imaging, College of Veterinary Medicine, Jeonbuk National University, Iksan, Republic of Korea; 2 Biosafety Research Institute and College of Veterinary Medicine, Jeonbuk National University, Iksan, Republic of Korea; 3 Sungsim Animal Medical Center, Daejeon, Republic of Korea

**Keywords:** congenital heart disease, atrial septal defect, ventricular septal defect, case report, echocardiography, Eisenmenger syndrome, shunt

## Abstract

Concurrent atrial septal defect (ASD) and ventricular septal defect (VSD) are rare in cats. A 7-month-old intact female Sphynx cat was presented for evaluation of respiratory distress and exercise intolerance. Thoracic radiographs showed generalized cardiomegaly and pulmonary infiltrates, consistent with cardiogenic pulmonary edema. Transthoracic echocardiography revealed an ostium secundum–type ASD and a perimembranous VSD, with structurally normal atrioventricular valves and annuli. These features supported the diagnosis of concurrent ASD and VSD, rather than an atrioventricular septal defect. Color Doppler echocardiography and agitated saline contrast studies confirmed bidirectional shunting at both defect sites. Additionally, echocardiographic findings were consistent with pulmonary hypertension. As Eisenmenger syndrome was suspected and the owner declined invasive intervention, surgical repair was not performed. Initial treatment with furosemide and supplemental oxygen led to clinical improvement. The owner later discontinued diuretics; however, the cat remained asymptomatic for several months. Cyanosis and erythrocytosis developed approximately 8 months after initial presentation. Follow-up thoracic imaging and Doppler echocardiography demonstrated reduced cardiac silhouette size without recurrent pulmonary edema, right-sided chamber enlargement with right ventricular hypertrophy, and decreased pulmonary-to-systemic flow ratio, consistent with progression of Eisenmenger physiology. Medical management with phlebotomy, sildenafil, clopidogrel, and oxygen supplementation provided temporary stabilization; however, the cat eventually died approximately 1 year after diagnosis, likely due to disease progression and inconsistent therapeutic compliance. Postmortem examination confirmed both septal defects and marked right ventricular hypertrophy, consistent with the ante-mortem imaging findings. Histopathological examination revealed pulmonary vascular remodeling and myocardial fibrosis, indicative of chronic pulmonary hypertension. This report represents the first documented case of concurrent ASD and VSD with Eisenmenger physiology in a cat, with long-term clinical follow-up and postmortem confirmation.

## 1 Introduction

Atrial septal defects (ASDs) and ventricular septal defects (VSDs) are among the most frequently diagnosed congenital cardiac anomalies in cats. A retrospective study reported that VSDs and ASDs accounted for approximately 50 and 10% of feline congenital cardiac defects, respectively (1). However, the simultaneous occurrence of both defects is rare. Although this combination has been frequently reported in humans (2), only isolated cases have been documented in cats (1, 3).

The presence of both an ASD and a VSD may represent either two distinct septal defects or a form of atrioventricular septal defect (AVSD). AVSDs are characterized by abnormalities involving both the atrioventricular septum and atrioventricular valves (4–6). Among AVSD subtypes associated with ASD, the primum-type defect near the atrioventricular junction is typical (7). When the atrioventricular valves and annuli are structurally normal, the defects are classified as concurrent ASD and VSD.

Septal defects permit abnormal communication between the systemic and pulmonary circulations. In severe cases, progressive pulmonary hypertension (PH) and Eisenmenger physiology may develop (8–13). The present report describes a cat with concurrent secundum-type ASD and perimembranous VSD that showed progressive hemodynamic deterioration associated with Eisenmenger physiology, documented through long-term clinical follow-up and confirmed by postmortem examination.

## 2 Case description

A 7-month-old intact female Sphynx cat weighing 2.8 kg was referred to our institution for evaluation of tachypnea and exercise intolerance. Physical examination revealed panting and pulmonary crackles on thoracic auscultation. Systolic blood pressure, measured using a Doppler device, was 115 mmHg, heart rate was 200 bpm, and rectal temperature was 38.8 °C.

Complete blood count results were within normal limits. Serum biochemistry revealed an elevated feline B-type natriuretic peptide concentration, whereas other parameters, including liver and renal values, were unremarkable.

**Figure 1:**
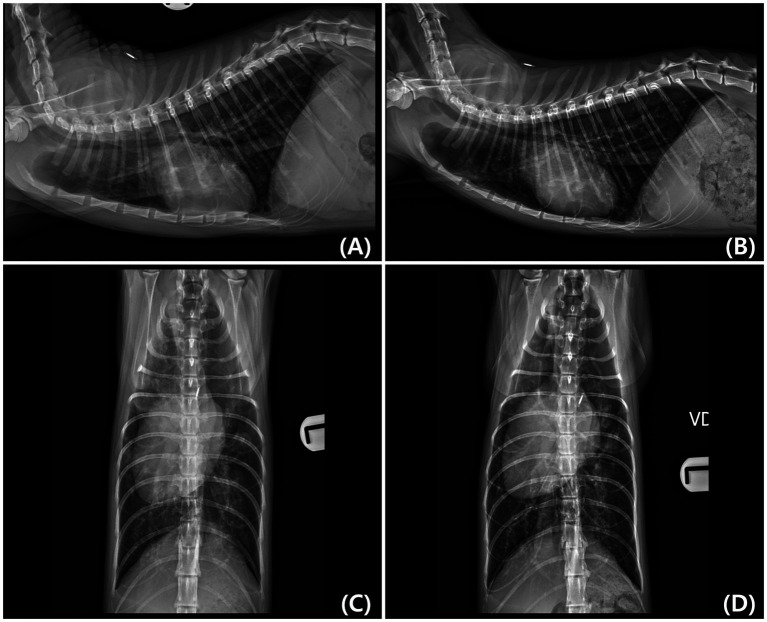
Right lateral and ventrodorsal (VD) thoracic radiographs obtained at initial diagnosis **(A,C)** and 8 months later **(B,D)**. **(A,C)** Initial radiographs show generalized cardiomegaly with rightward displacement of the cardiac silhouette and pulmonary infiltrates. **(B,D)** Follow-up radiographs demonstrate a reduction in cardiac silhouette size (Vertebral heart score [VHS] decreased from 8.7 to 8.2) and resolution of pulmonary edema. A slight rightward rotation of the thoracic spine is visible in **(D)**, which may have influenced the apparent width of the cardiac silhouette.

Thoracic radiographs demonstrated generalized cardiomegaly with a vertebral heart score (VHS) of 8.7 and thoracic width-to-T4 ratio of 4.4, both exceeding the normal feline reference ranges (RR) (VHS: 7.5 ± 0.3, width-to-T4 ratio: 3.4 ± 0.25) (14). Additional radiographic findings included an enlarged cardiac silhouette spanning three intercostal spaces, rightward displacement of the cardiac silhouette, alveolar pulmonary infiltrates in both the cranial and caudal lung lobes, and hepatomegaly (Figures 1A,C). Thoracic radiographs were acquired using a digital system (Ecoray, Seoul, Korea), and images were stored in the DICOM format.

Electrocardiography revealed a deep S-wave in lead II and a positive QRS complex only in lead aVR, with all other leads showing negative deflections, indicating an extreme right axis deviation.

**Figure 2:**
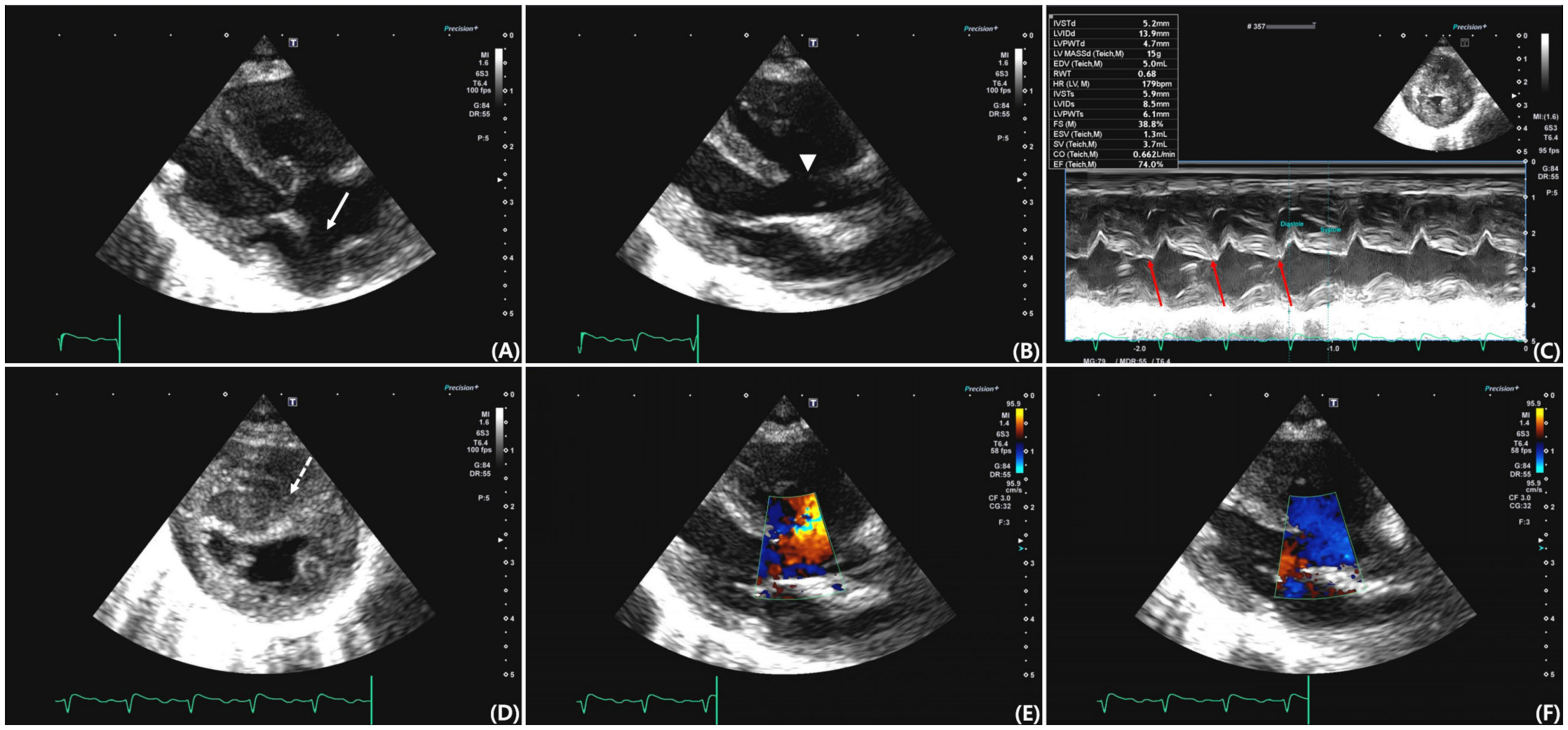
Transthoracic echocardiographic images from various views. **(A)** Right parasternal long-axis four-chamber view showing an atrial septal defect (ASD) located at the region of the fossa ovalis (arrow). **(B)** Right parasternal long-axis five-chamber view showing a perimembranous ventricular septal defect (VSD) (arrowhead). **(C)** M-mode image demonstrating paradoxical septal motion (red arrow). **(D)** Right parasternal short-axis view at the level of the papillary muscles showing septal flattening and concentric right ventricular hypertrophy (dashed arrow). **(E)** Color Doppler image obtained during systole showing left-to-right shunt flow across the ventricular septal defect. **(F)** Color Doppler image obtained during diastole demonstrating right-to-left shunt flow across the same defect.

Transthoracic echocardiography was performed using a Canon Aplio i800 ultrasound system (Canon Medical Systems, Tokyo, Japan) equipped with a sector transducer (3.5–12 MHz). An ostium secundum–type ASD located within the fossa ovalis and a perimembranous VSD were identified. Maximal end-diastolic diameters measured in B-mode were 4.55 and 6.70 mm for the ASD and VSD, respectively. The atrioventricular annuli were separated, and both the mitral and tricuspid valves appeared to be structurally normal (Figures 2A,B). The pulmonary valve appeared morphologically normal, with no evidence of an overriding aorta.

M-mode imaging demonstrated paradoxical septal motion (Figure 2C), and right ventricular (RV) concentric hypertrophy was observed in the right parasternal short-axis view (Figure 2D). The left atrium was mildly enlarged, with a left atrial-to-aortic root ratio (LA/Ao) of 1.58 (RR: 1.3–1.5) (15), and tricuspid regurgitation velocity was markedly elevated (5.38 m/s; RR: 0.88–2.71 m/s) (16). The right atrium was dilated, and right pulmonary artery distensibility was 10%, a value below those reported in healthy cats, although no established reference range is available (17). The pulmonary valve acceleration time was shortened (AT, 50 ms; RR: 59 ± 9 ms), and the AT/ET ratio was reduced (0.19; RR: 0.41 ± 0.06) (16). Tissue Doppler imaging revealed decreased early (E′: 4.7 cm/s; RR: 5.0–9.0 cm/s) and late (A′: 4.5 cm/s; RR: 6.0–8.0 cm/s) diastolic velocities at the septal annulus (18), an increased isovolumetric relaxation time (IVRT: 110 ms; RR: 45.7 ± 3.3 ms) (19), and a reduced S′ wave (5.1 cm/s; RR: 5.1–9.1 cm/s) (16). The E/E' ratio was 11.49, and the tricuspid annular plane systolic excursion was reduced (3.9 mm; RR: 9.1 ± 1.4 mm). These findings are consistent with impaired left ventricular relaxation and decreased RV systolic function.

Color Doppler and echocardiography demonstrated bidirectional shunting flow across both septal defects. A left-to-right shunt was observed during systole (Figure 2E) and right-to-left shunt was observed during diastole (Figure 2F). An agitated saline contrast study revealed the presence of microbubbles in the LA, followed by bubbles in the aorta, confirming right-to-left shunting. Surgical correction was not performed because Eisenmenger syndrome was suspected, and the owner declined invasive intervention.

Initial medical management included subcutaneous furosemide injection (2 mg/kg) and supplemental oxygen therapy administered via an isolated oxygen chamber. After 4 h, furosemide (1 mg/kg) was administered intravenously. Oral furosemide (1.5 mg/kg twice daily) and clopidogrel (18.75 mg/cat once daily) were continued for 2 months after initial presentation, whereby the cat remained clinically stable. However, after this period, the owner discontinued treatment without veterinary consultation.

The cat developed cyanosis 8 months after initial presentation. Follow-up thoracic radiography revealed a reduction in cardiac silhouette size (VHS decreased from 8.7 to 8.2) and no recurrence of pulmonary edema (Figures 1B,D). Additionally, echocardiography revealed right atrial enlargement and RV hypertrophy compared with that of the initial examination, and Doppler echocardiography showed a decreased pulmonary-to-systemic flow ratio ([Qp/Qs]: 1.28 → 0.67), suggesting reduced pulmonary perfusion. Peripheral oxygen saturation (SpO₂) measured from the tail decreased from approximately 90 to 80%, and hematocrit was markedly elevated (58.2%; RR: 30.3–52.3%), consistent with chronic hypoxemia. Furthermore, serum blood urea nitrogen increased (42.2 mg/dL; reference, 17.6–32.8 mg/dL), whereas other renal parameters remained within normal limits.

Phlebotomy and intravenous fluid therapy were initiated to reduce hematocrit to below 50–55%. Oral sildenafil (2 mg/kg twice daily) and clopidogrel (18.75 mg/cat once daily) were administered, along with continued oxygen supplementation and strict cage rest. Clinical signs improved, and for the following 4 months, the cat received regular medication and was placed in a custom-made oxygen chamber three times daily or as needed during episodes of cyanosis. Despite medical management, the cat died at home approximately 1 year after initial presentation.

**Figure 3:**
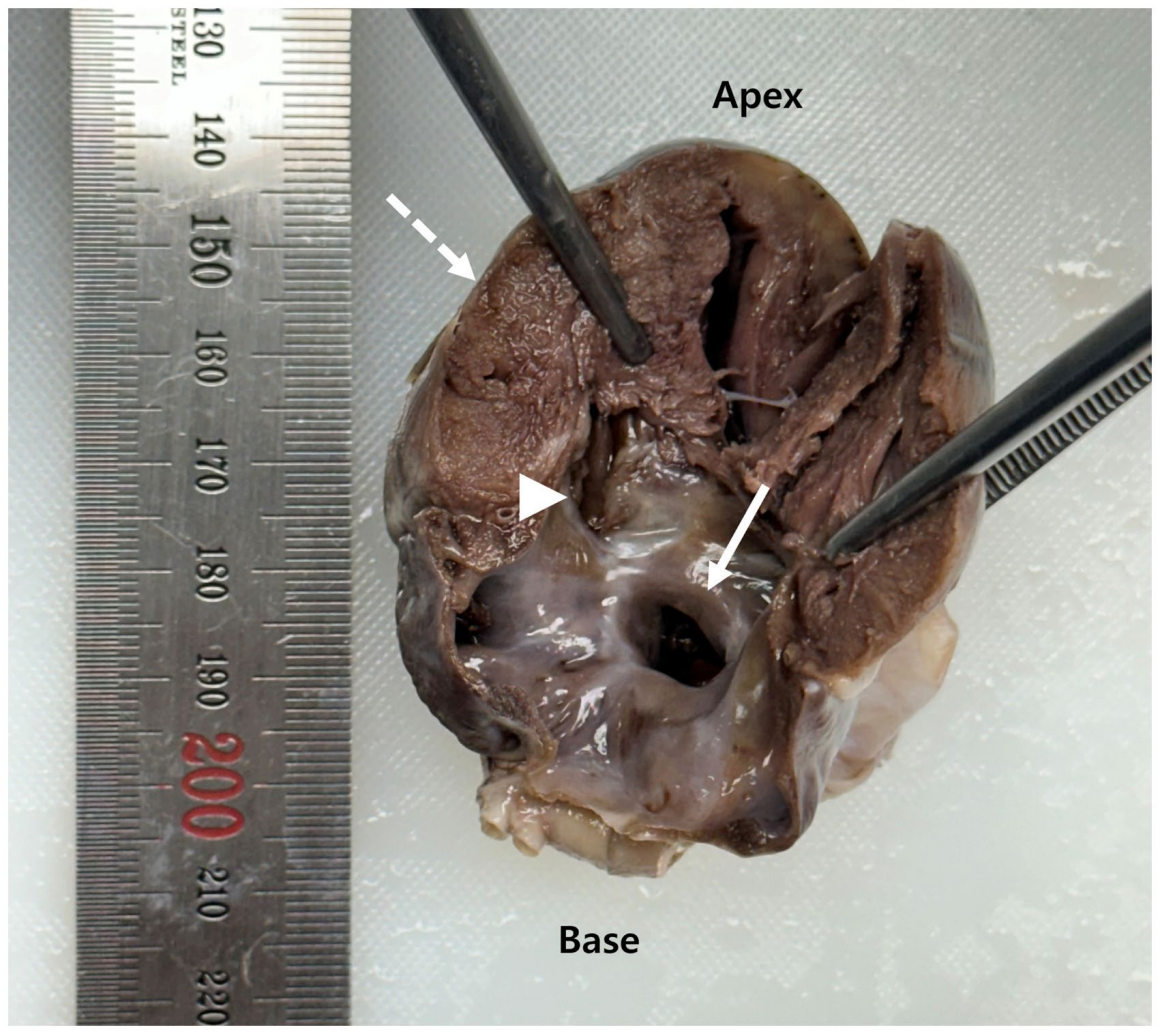
Gross longitudinal section of the heart obtained at necropsy. The arrow indicates an atrial septal defect (ASD) located at the fossa ovalis. The arrowhead identifies a perimembranous ventricular septal defect (VSD). The dashed arrow highlights concentric right ventricular myocardial hypertrophy.

Subsequently, a necropsy was performed at our institution, confirming the presence of both septal defects. The ASD was located in the fossa ovalis, and the VSD was perimembranous. The atrioventricular annuli were intact, and both mitral and tricuspid valves appeared structurally normal. The pulmonary valves were morphologically unremarkable, with no evidence of stenosis or dysplasia. Gross examination revealed marked RV myocardial hypertrophy (Figure 3).

**Figure 4:**
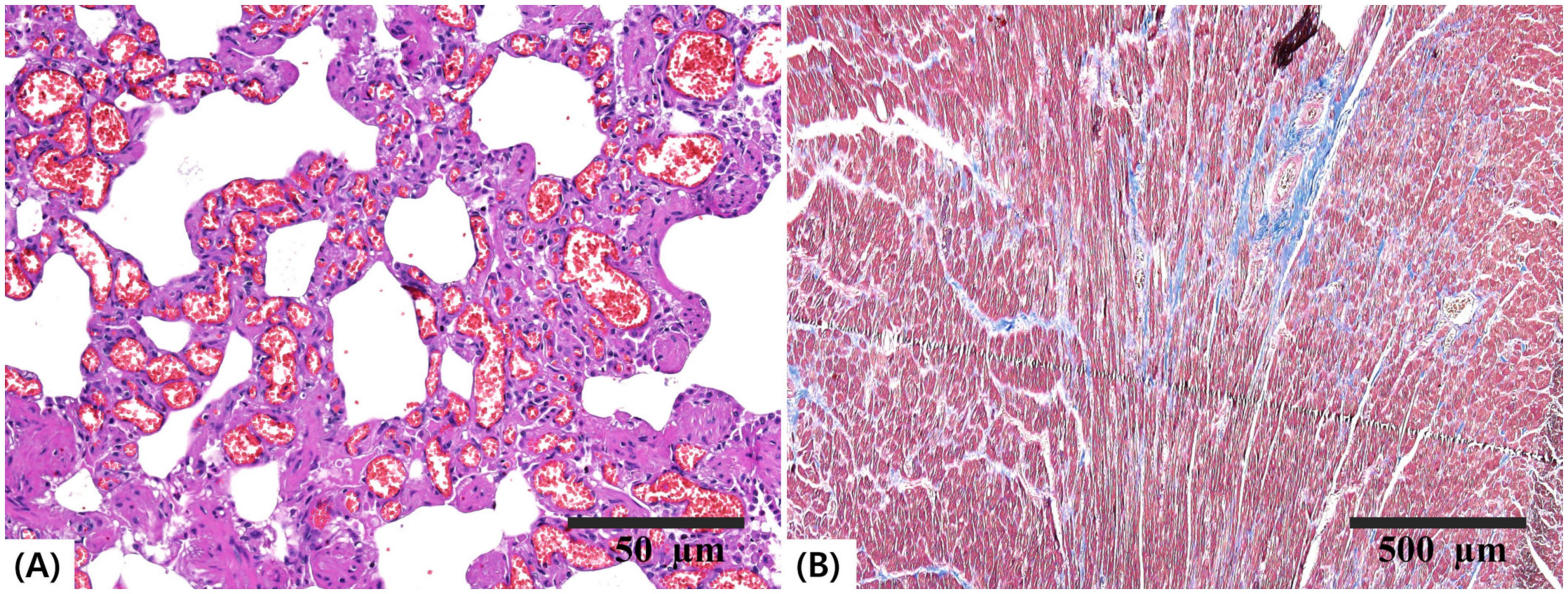
Histopathological sections of the lung and heart. **(A)** Lung tissue stained with hematoxylin and eosin (H&E; scale bar = 50 μm) showing diffuse capillary ectasia and perivascular inflammatory cell infiltration. **(B)** Right ventricular myocardium stained with Masson’s trichrome (scale bar = 500 μm) demonstrating interstitial fibrosis.

Histopathological evaluation of the lungs revealed diffuse capillary ectasia and perivascular infiltration of inflammatory cells (Figure 4A). RV myocardial tissue showed interstitial fibrosis, which was confirmed histologically via Masson’s trichrome staining (Figure 4B).

## 3 Discussion

Thoracic radiographic findings at presentation were consistent with cardiogenic pulmonary edema, as supported by clinical signs and improvement following diuretic therapy (20, 21). Electrocardiographic findings revealed extreme right axis deviation, indicative of RV hypertrophy, which corresponded with radiographic evidence of right-sided cardiac enlargement (12, 13). Echocardiographic evaluation revealed two distinct septal defects without abnormalities at the atrioventricular junction, such as a common atrioventricular valve or malformed annuli, thereby excluding an AVSD. Although the coexistence of large atrial and ventricular septal defects may resemble AVSD on imaging, the presence of clearly separated annuli and structurally normal mitral and tricuspid valves supports the classification of these lesions as concurrent ASD and VSD. These features are inconsistent with AVSD, which typically involves a primum-type ASD accompanied by atrioventricular valve malformations or annular defects (4–7). In addition, the absence of an overriding aorta and presence of a morphologically normal pulmonary valve excluded pentalogy of Fallot as a differential diagnosis (22). Additional echocardiographic findings, including right-sided chamber enlargement, reduced right pulmonary artery distensibility, and paradoxical septal motion, indicated the presence of PH and chronic pressure overload (8, 10, 12, 13, 23). Bidirectional shunt flow was confirmed through an agitated saline contrast study and color Doppler imaging. Surgical intervention was contraindicated owing to the presence of Eisenmenger syndrome on echocardiography (23, 24), and the owner declined invasive intervention. Therefore, the cat was managed conservatively with furosemide, clopidogrel, and oxygen supplementation.

This conservative approach initially led to a temporary resolution of the clinical signs, supporting the interpretation that pulmonary edema was present at that time. Although bidirectional shunting was evident, the occurrence of pulmonary edema suggests that left-to-right flow remained predominant, which may explain the observed pulmonary overcirculation (24–26). However, pulmonary hemorrhage or alveolar infiltrates secondary to severe PH could not be definitely excluded. Additionally, differential diagnoses, including pulmonary veno-occlusive disease and pulmonary capillary hemangiomatosis, have been reported to produce similar radiographic findings, typically in the absence of marked left-sided cardiac enlargement (27–29). Although the owner discontinued diuretic therapy without veterinary consultation after the initial improvement, the cat remained asymptomatic for 2 months, and no evidence of pulmonary edema was observed over the subsequent 6 months. This apparent stabilization should not be interpreted as disease resolution but rather may reflect a shift toward increased right-to-left shunting, resulting in reduced pulmonary perfusion and progressive PH despite apparent clinical improvement (24). Approximately 8 months after the initial presentation, the cat returned with cyanosis and marked erythrocytosis accompanied by reduced Qp/Qs and SpO₂, which is consistent with chronic hypoxemia and progression of Eisenmenger physiology (12, 13, 23, 30). Echocardiographic findings, including right atrial enlargement and RV hypertrophy, further indicated sustained PH and right-sided pressure overload (31, 32). Although the development of Eisenmenger physiology is generally associated with a poor prognosis, survival outcomes vary depending on the degree of pulmonary vascular remodeling and shunt flow balance. In some cases, right-to-left shunting may persist long-term under medical management (24, 30, 33–36), and survival beyond 9 years has been reported in a dog with Eisenmenger physiology secondary to a VSD (36). Additionally, shunt reversal from right-to-left to left-to-right has been observed in response to pulmonary vasodilator therapies, such as sildenafil (24). These observations suggest that appropriate medical interventions play a critical role in modulating disease progression. Following the onset of right-to-left shunting, the cat was treated using phlebotomy, sildenafil, clopidogrel, and oxygen supplementation. These interventions have been reported to mitigate clinical manifestations associated with right-to-left shunting, including polycythemia, hypoxemia, and thromboembolic risk (24, 30, 33, 36–38). Although a temporary reduction in cyanotic episodes was observed after treatment initiation, the cat ultimately died approximately 1 year after the initial diagnosis, most likely due to progressive PH. Additionally, limited follow-up and inconsistent treatment may have contributed to the clinical outcome (36). Cats with Eisenmenger syndrome should be monitored for disease progression and deterioration of clinical signs. However, the effects of stress caused by re-examination should also be considered.

Necropsy confirmed the presence of two septal defects and morphologically normal atrioventricular annuli and valves, consistent with the echocardiographic diagnosis of concurrent ASD and VSD. RV myocardial hypertrophy has also been observed, indicating sustained pressure overload (31, 32). Histopathological analysis revealed pulmonary vascular remodeling with fibrotic changes consistent with chronic PH (12, 39). Moreover, RV interstitial fibrosis was observed, indicating prolonged right-sided pressure overload (40). These postmortem findings confirmed the hemodynamic burden suggested by ante-mortem imaging. The presence of two separate septal defects likely increased total shunt volume, promoting excessive pulmonary perfusion. Chronic overcirculation may induce endothelial injury and trigger fibrotic remodeling within the pulmonary vasculature (41, 42). Over time, these changes lead to an irreversible elevation of pulmonary vascular resistance and eventual shunt reversal (42). Concurrent ASD and VSD may have accelerated this transition, contributing to the rapid progression of Eisenmenger physiology.

One limitation of this case is the lack of continuous treatment and monitoring due to the owner’s limited cooperation, which restricted opportunities for screening tests and timely therapeutic adjustment. This limitation may have contributed to the clinical course; however, prognosis in such patients is multifactorial, and the influence of follow-up should be interpreted with caution.

This case report describes a Sphynx cat with concurrent secundum-type ASD and perimembranous VSD, which exhibited progression of Eisenmenger physiology. The diagnosis was confirmed by clinical follow-up, echocardiography, necropsy, and histopathology. Although these congenital defects have been reported individually, their concurrent occurrence is rare. To the best of our knowledge, this is the first case of long-term clinical follow-up and postmortem confirmation of Eisenmenger physiology in a cat.

## Data Availability

The original contributions presented in the study are included in the article/Supplementary material, further inquiries can be directed to the corresponding author.

## Supplementary material

SUPPLEMENTARY FIGURE 1 Echocardiographic images used to calculate the pulmonary-to-systemic flow ratio (Qp/Qs) at initial **(A,C,E,G)** and follow-up **(B,D,F,H)** examinations. **(A,B)** Pulmonary artery diameter measured at the right ventricular outflow tract during systole. **(C,D)** Velocity–time integral (VTI) of pulmonary flow obtained from the same site. **(E,F)** Aortic diameter measured at the left ventricular outflow tract during systole. **(G,H)** VTI of aortic flow obtained from the left ventricular outflow tract. Cross-sectional areas and VTIs were used to calculate Qp/Qs ([PA area × PA VTI] / [Ao area × Ao VTI]).

SUPPLEMENTARY VIDEO 1 Agitated saline contrast study demonstrating bidirectional shunting through both septal defects. Cine loop of the heart showing immediate appearance of several microbubbles in the left atrium and left ventricle after their entry into the right atrium and right ventricle, indicating pronounced right-to-left shunting across both the atrial and ventricular septal defects.

SUPPLEMENTARY VIDEO 2 Agitated saline contrast study demonstrating bidirectional shunting through both septal defects. Cine loop of the descending aorta showing subsequent visualization of microbubbles, further confirming right-to-left shunting at the systemic level.
