# Supplementary figures and images for: Case Report: Concurrent atrial and ventricular septal defect in a young Sphynx cat
Source: Front Vet Sci. 2025 Nov 19;12:1684236. doi: 10.3389/fvets.2025.1684236 (PMC12673618; doi:10.3389/fvets.2025.1684236)

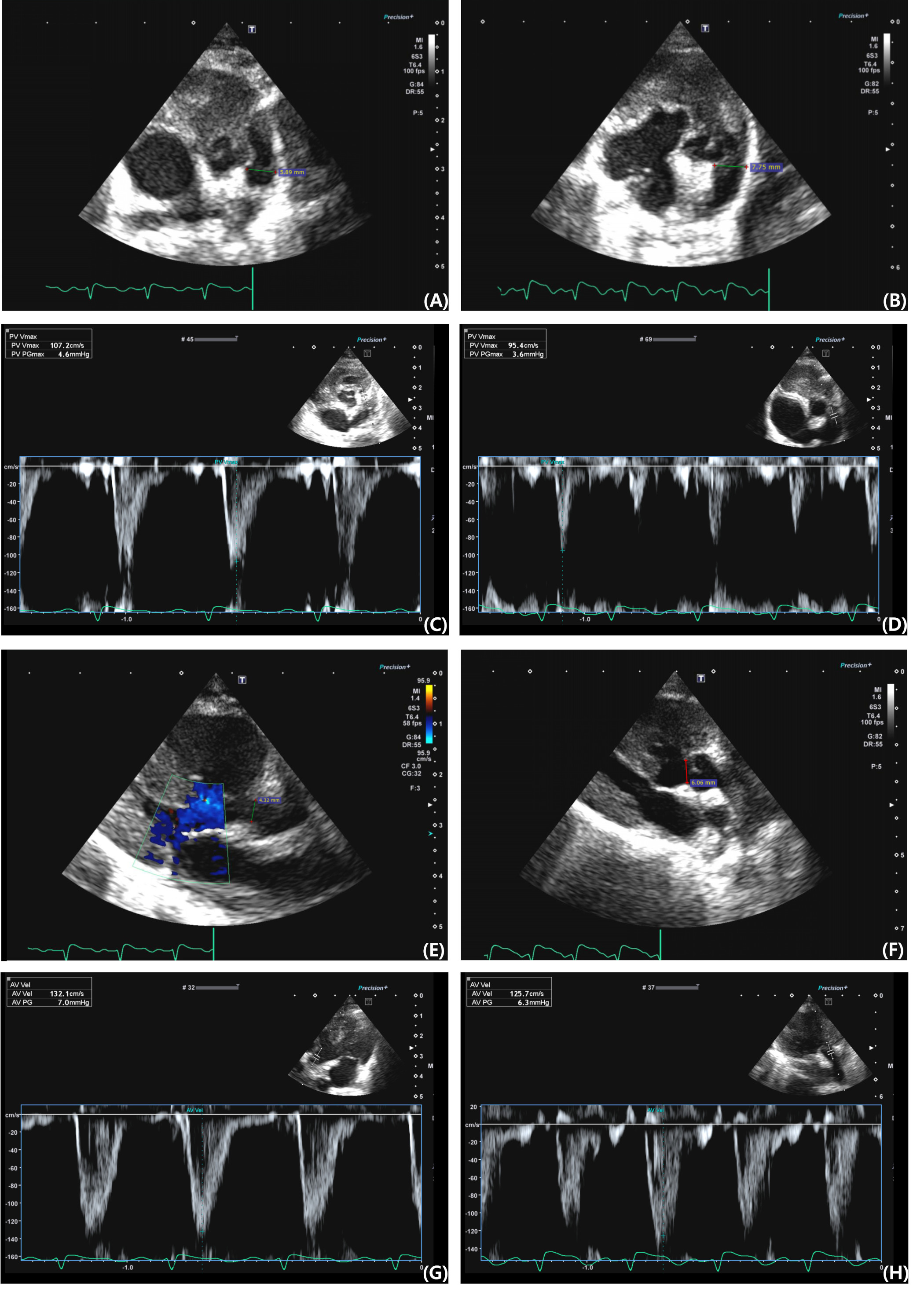

Supplement: SUPPLEMENTARY FIGURE 1 — Echocardiographic images used to calculate the pulmonary-to-systemic flow ratio (Qp/Qs) at initial (A,C,E,G) and follow-up (B,D,F,H) examinations. (A,B) Pulmonary artery diameter measured at the right ventricular outflow tract during systole. (C,D) Velocity–time integral (VTI) of pulmonary flow obtained from the same site. (E,F) Aortic diameter measured at the left ventricular outflow tract during systole. (G,H) VTI of aortic flow obtained from the left ventricular outflow tract. Cross-sectional areas and VTIs were used to calculate Qp/Qs ([PA area × PA VTI] / [Ao area × Ao VTI]). [file Image_1.jpeg]
