# Supplementary material for: Case Report: Concurrent atrial and ventricular septal defect in a young Sphynx cat
Source: Front Vet Sci. 2025 Nov 19;12:1684236. doi: 10.3389/fvets.2025.1684236 (PMC12673618; doi:10.3389/fvets.2025.1684236)
Supplement: Supplementary file 4 [file Table_1.docx]

1. **A table showcasing a timeline with relevant data from the episode of care**

| \| Timepoint \| Event/Findings \| \| --- \| --- \| \| Initial Presentation (7 months old) \| Tachypnea and exercise intolerance; imaging and echocardiography confirmed ASD and VSD; elevated BNP; furosemide and supplemental oxygen initiated. \| \| First 2 months post-diagnosis \| Maintained on oral furosemide and clopidogrel; clinically stable. \| \| After 2 months \| Owner discontinued medication \| \| 8 months after initial presentation \| Cyanosis and chronic hypoxemia; Phlebotomy, IV fluids, sildenafil, and clopidogrel were started. \| \| Following 4 months \| Regular medication and oxygen therapy using a custom-made chamber. \| \| 1 year \| Died at home despite medical management. \| \| Postmortem \| Confirmed ASD, VSD, RV hypertrophy, pulmonary capillary ectasia, myocardial fibrosis. \| |
| --- | --- | --- | --- | --- | --- | --- | --- | --- | --- | --- | --- | --- | --- | --- | --- | --- |
